# Wheelchair as a nexus: understanding stigma in older adults with stroke in China

**DOI:** 10.3389/fmed.2025.1742686

**Published:** 2026-01-13

**Authors:** Qingqing Chu, Jing Zhang, Min Xi, Miaosen Liang, Xinyue Wang, Donggui You, Xinxin Yu

**Affiliations:** 1 School of Fine Arts, Hubei University of Arts and Sciences, Xiangyang, Hubei, China; 2 Malaysian Research Institute on Ageing, Universiti Putra Malaysia, Serdang, Malaysia; 3 Xiangyang Central Hospital, Affiliated Hospital of Hubei University of Arts and Science, Xiangyang, Hubei, China

**Keywords:** assistive device design, older adults, rehabilitation, stigma, stroke, wheelchair

## Abstract

**Purpose:**

After a stroke, older adults often face compounded stigma due to disability and aging, which can hinder their rehabilitation engagement and community participation. As wheelchairs are central to long-term stroke management, their design can either reinforce or mitigate such stigma. This study explores how wheelchair design attributes influence stigma among Chinese older adults with stroke and how these perceptions affect rehabilitation adherence and social reintegration.

**Method:**

A qualitative case study using semi-structured interviews was conducted with 15 older adults with stroke, 20 caregivers and rehabilitation stakeholders, and 24 members of the general public in Hubei, China. Data were thematically analyzed within an interdisciplinary rehabilitation context to identify how wheelchair-related meanings shape psychosocial and behavioral outcomes.

**Results:**

Five interrelated themes emerged: symbolic impact on personal identity, perceived visual aesthetics, adaptability across daily living contexts, autonomy and control in wheelchair use, and ease of operation in wheelchair maneuvering. Findings show that wheelchair design plays a dual role in the stigmatization process-it may serve as a visible marker of dependence or, if optimally designed, promote autonomy, dignity, and social acceptance.

**Conclusion:**

Wheelchair characteristics influence not only self-stigma and social perception but also rehabilitation adherence and quality of life among older adults with stroke. Addressing stigma through design requires integrating symbolic, sociocultural, and psychological dimensions into assistive device development. These findings highlight the need for interdisciplinary collaboration across design, rehabilitation, and integrated care networks to ensure equitable, stigma-free assistive solutions that enhance the well-being of older adults with stroke.

## 1 Introduction

China is undergoing one of the world’s most rapid aging processes, accompanied by increasing health challenges among older adults (1). The burden of stroke remains high in China, which has one of the highest stroke mortality rates globally (58). For many older adults with stroke, especially older individuals, wheelchairs serve as essential assistive devices that enable mobility and help maintain independence in daily living (2). However, older adults with stroke are often perceived as a burden to society, suffering from discrimination and marginalization due to the stigma associated with both aging and disability (3, 4). These stigmatizing perceptions can hinder rehabilitation, reduce social participation, and exacerbate psychological distress (5, 6).

Stigma is a social process characterized by exclusion, rejection, and devaluation, arising from the perception or experience of negative social evaluations toward certain individuals or groups (7, 8). In the context of disability and aging, stigma can be both externally imposed by society and internally adopted by individuals, influencing self-esteem and identity (9, 10). Health-related stigma, closely linked to ageism, imposes additional psychological burdens on older adults with stroke, hindering recovery and diminishing quality of life (11–13). Older adults with stroke must therefore navigate not only the physical challenges of their condition but also the social stigma tied to age and disability, further intensifying psychological distress (14, 15). Consequently, the symbolic and functional demands associated with wheelchair use can intensify existing stigma among older adults with stroke, by making physical limitation visible and shaping social perceptions of dependency.

Assistive technologies provide important functional benefits but are often perceived as visible symbols of incapacity or loss, reinforcing negative stereotypes and social labeling (3, 16, 17). The stigma associated with assistive devices may reduce social participation and hinder the community integration of older adults (18, 19). Addressing these barriers requires an interdisciplinary approach that bridges design, rehabilitation medicine, and community-based primary care.

Design plays a critical role in shaping how assistive technologies are perceived and experienced. Product semantics theory suggests that the form, color, and material of objects convey symbolic meanings that shape users’ emotional responses and self-perception (20, 21). Product design can either reinforce stigma or serve as a means of reducing it (22). For assistive devices, overly medicalized or purely functional aesthetics can heighten feelings of vulnerability and dependency among users, while also eliciting negative judgments from caregivers and bystanders (22–24). Thus, the design of wheelchairs is not merely a technical or functional issue-it is deeply intertwined with users’ sense of identity, personal dignity, and capacity for social engagement.

Recent studies have explored stigma, aesthetics, and user acceptance in assistive and rehabilitation technologies, highlighting the importance of appearance, usability, and social meaning in shaping device acceptance and identity reconstruction. The public visibility of medical-looking devices often exposes users to social scrutiny, stares, and subtle forms of discrimination, further intensifying stigma and discouraging consistent use in public spaces (25). In many cultural contexts, wheelchairs and other visible assistive devices are semantically interpreted as symbols of weakness, illness, and dependency, which can trigger discomfort and a perceived loss of dignity among older users (3). Evidence suggests that modern, stylish, and personalized designs increase users’ willingness to adopt assistive devices, whereas poorly designed or overly medicalised products may reinforce feelings of stigma, emotional distancing, and lowered self-esteem (26). These findings underscore the importance of situating assistive technology (AT) design within social and cultural contexts. Given China’s rapidly aging population and high stroke prevalence, there is a pressing need to translate existing design and rehabilitation research into culturally relevant and usable assistive solutions.

The purpose of this study is to investigate how wheelchair design shapes the stigma experienced by older adults with stroke in China. The study focuses on the long-term post-stroke rehabilitation period. It examines key wheelchair design features, including visual form, usability, maneuverability, and other relevant characteristics, and investigates how these features influence outcomes such as perceived stigma, social participation, and use adherence among older adults with stroke.

Building on empirical evidence, this study identifies critical design features and investigates stigma associated with wheelchair use within the Chinese cultural context. It clarifies how specific design attributes relate to psychosocial outcomes. Furthermore, it proposes socio-emotional design principles along with practical guidance for designers and clinicians, and outlines methodological directions to support longitudinal and cross-cultural research on AT acceptance and stigma reduction.

## 2 Materials and methods

### 2.1 Research design

**Figure 1:**
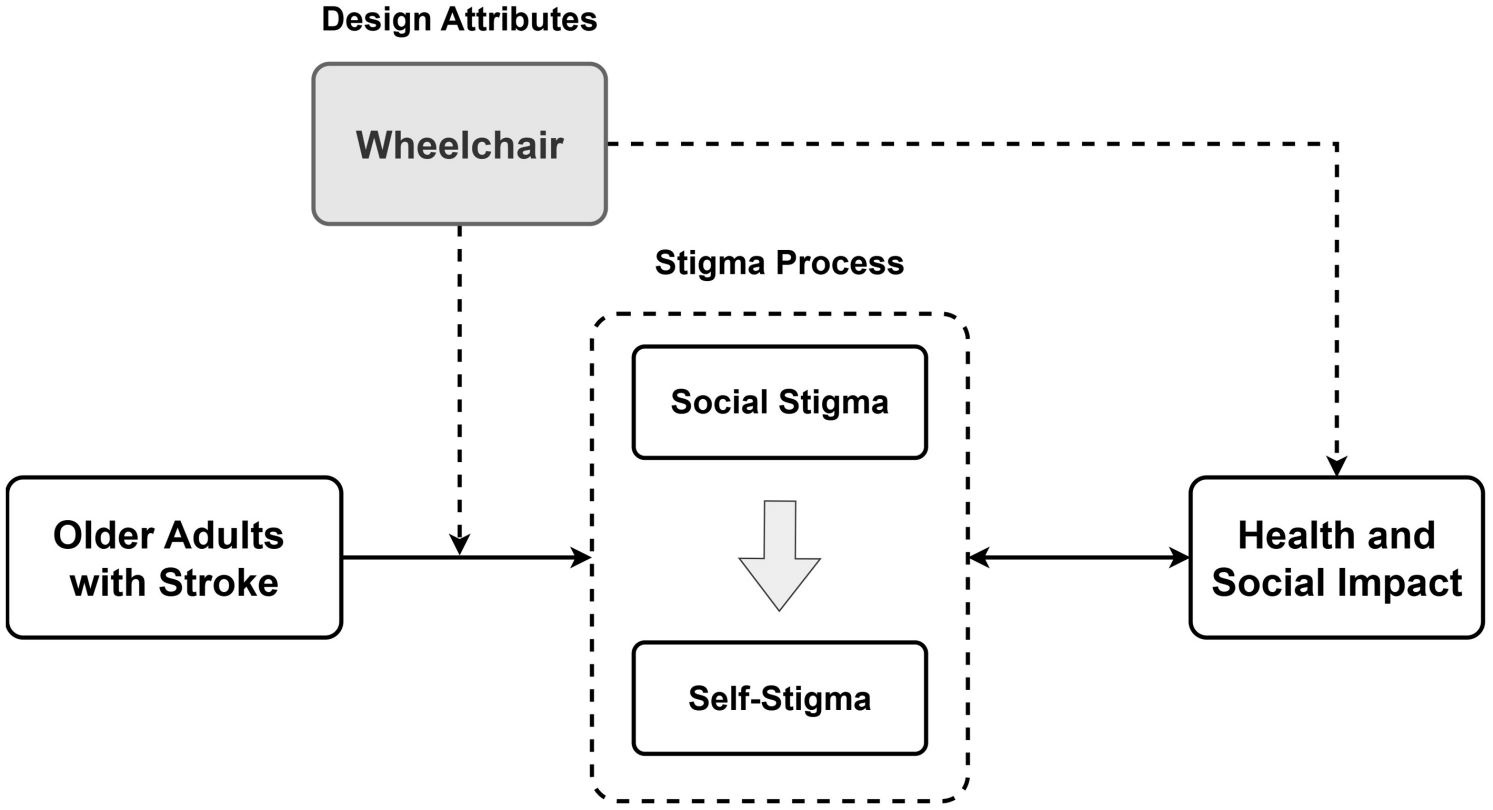
Conceptual framework of moderating factors related to the stigma of wheelchair use among older adults with stroke.

A conceptual framework was developed by integrating the Health Stigma and Discrimination Framework (13, 27, 55) with a model illustrating the effects of stigma processes on health outcomes. The framework was primarily theory-driven, linking the stigma process to the symbolic meanings embodied in assistive product design (see Figure 1). Guided by this conceptual framework, the study adopted a case study approach to explore the relationship between wheelchair design and stigma among older adults with stroke (57). The case study method was selected because the phenomenon being investigated can only be understood within a real-world context that includes rehabilitation settings, caregiving routines, and everyday interactions. This approach also enables a holistic examination of the issue through multiple data sources and diverse stakeholder perspectives. It not only helps identify key wheelchair design attributes associated with stigma but also provides deeper interpretive insights into how these attributes influence the lived experiences of older adults with stroke.

Semi-structured interviews and non-participant observations were employed as complementary data collection techniques, allowing for the collection of diverse stakeholder perspectives and data triangulation. The conceptual framework guided the development of the semi-structured interview by defining three key domains of inquiry: (1) Lived experiences of stigma in everyday life, (2) Perceptions of wheelchair design features such as appearance, comfort, and usability, and (3) Stakeholder perspectives on design improvements to reduce stigma and support rehabilitation. By explicitly connecting theoretical constructs to data collection, this framework provided both a conceptual foundation and a practical structure for the interviews. In addition, the development of the interview guide was informed by insights gained from the literature review, early field observations, the conceptual framework of stigma and well-being, and preliminary consultations with rehabilitation experts (see Appendix 1). To refine the structure and clarity of the interview questions, a pilot study was conducted prior to the formal data collection. Three individuals participated in the pilot phase: an older adult with stroke and his caregiver from Xiangyang Shouxing Nursing Home, and a community resident from Xiangyang Shanshui Home Community. Feedback and issues identified during the pilot interviews were analyzed to optimize the relevance, sequencing, and comprehensibility of the questions. This iterative process ensured the rigor and quality of the subsequent formal interviews and enhanced the reliability of the collected data.

### 2.2 Participants

Purposive sampling was employed to recruit participants who could provide rich, relevant insights aligned with the study’s objectives (59, 60). Recruitment was facilitated through recommendations from social workers, hospital rehabilitation therapists, and nursing home caregivers.

All participants met the inclusion and exclusion criteria established for this study (see Table 1). The research was conducted in several urban areas of Hubei Province, China. All older adult participants used manual wheelchairs, as these represent the most common and affordable mobility aids within the local context and are highly visible in both community and institutional care settings. Recruitment continued until data saturation was achieved, which refers to the point when no new insights emerged about the stigma experienced by older adults with stroke in relation to wheelchair use.

**Table 1:** Inclusion and exclusion criteria of the target population in the interview.

Target population	Inclusion criteria	Exclusion criteria
Older adults with stroke	1. Chinese individuals aged 60–80 years old. 2. Stroke patients in the subacute phase (2 months to 6 months after the stroke onset) or the chronic phase (more than 6 months after the stroke onset). 3. Currently using or having experience of using wheelchairs after a stroke. 4. Able to communicate verbally, with sufficient cognitive ability to provide informed responses. 5. Willingness to participate in the study. 6. Consent from the caregivers or family members of the patients.	1. Chinese individuals aged<60 or>80. 2. Stroke patients with severe symptoms or complications post-stroke (e.g., swallowing disorders, advanced dementia, or severe mobility limitations) 3. Stroke patients unwilling to continue participating during the study. 4. Occurrence of stressful events such as death of family members, accident or new illness.
Members of the general public	1. Healthy individuals who regularly encounter or interact with post-stroke wheelchair users (e.g., in public spaces or neighbourhoods). 2. No known history of neurological or psychiatric disorders. 3. Willingness to participate in the study.	1. Unwilling to continue participation during the study.
Caregiver and nurse	1. Being the primary caregiver or providing direct nursing care for a minimum of 2 years. 2. Having experience in providing care for older adults with stroke 60–80 years old. 3. A willingness to participate.	1. Unwilling to continue participation during the study.
Rehabilitation physician and therapist	1. Licensed rehabilitation physician or therapist with a minimum of 2 years of professional experience. 2. Have experience in rehabilitation treatment for older adults with stroke 60–80 years old. 3. A willingness to participate.	1. Unwilling to continue participation during the study.
Family member of older adults with stroke	1. Immediate family members with caregiving experience for older adults with stroke relatives aged 60–80 years. 2. Willingness to participate in the study.	1. Unwilling to continue participation during the study.

Participants were divided into three primary groups: (1) older adults with stroke who were currently using or had previously used a manual wheelchair, providing firsthand accounts of stigma and lived experience; (2) stakeholders, including caregivers, rehabilitation therapists, nurses, and physicians, who offered professional perspectives on wheelchair use and its social implications in post-stroke care; and (3) members of the general public, including family members and community residents, who reflected broader societal perceptions and attitudes toward wheelchair users. In the proposed conceptualization, stigma surrounding wheelchair use among post-stroke older adults is understood as a relational and socio-symbolic process involving not only stroke survivors and caregivers, but also community bystanders who participate in the production and circulation of meaning in everyday social environments. Members of the general public were therefore included as an analytic group to capture how wheelchair use is interpreted, evaluated, and symbolically framed in routine social encounters.

Based on the sampling framework, participant recruitment and screening proceeded as follows. 40 older adults with stroke, 36 stakeholders, and 39 members of the general public were initially recruited. After screening and excluding those who did not meet the eligibility standards or withdrew consent, the final sample consisted of 59 participants. In total, 15 older adults with stroke, 20 stakeholders, and 24 members of the general public (including nine family members) participated in the study (see Tables 2–4 for participant details).

**Table 2:** The profile of interviewed older adults with stroke.

Participants	Sex	Age	Education	Living situation	Stroke subtype	Interview duration (min)
SA1	Male	80	High school	Nursing home	IS	25
SA2	Male	73	Primary school	Nursing home	HS	27
SA3	Male	61	Primary school	Nursing home	HS	31
SA4	Male	72	High school	Nursing home	IS	31
SA5	Male	74	College	Nursing home	IS	32
SA6	Male	76	College	Nursing home	IS	34
SA7	Female	63	Primary school	Nursing home	IS	26
SA8	Male	79	High school	Nursing home	IS	28
SA9	Female	65	Middle school	Community	IS	25
SA10	Female	68	Primary school	Community	IS	25
SA11	Male	61	Middle school	Community	IS	32
SA12	Male	72	Middle school	Community	HS	30
SA13	Female	70	Primary school	Community	IS	31
SA14	Male	72	High school	Community	HS	27
SA15	Male	60	Primary school	Community	HS	32

SA = older adults with stroke. Interview duration for older adults with stroke ranged from approximately 25 to 34 min.

**Table 3:** The profile of stakeholders in the interview.

Participants	Sex	Age	Education	Occupation	Work experience	Interview duration (min)
RN1	Female	26	College	Nurse	4	43
PR1	Male	45	Postgraduate	Rehabilitation physician	10	46
RN2	Female	29	College	Nurse	5	42
RN3	Female	41	College	Nurse	8	40
PR2	Male	34	Postgraduate	Rehabilitation physician	5	47
RT1	Female	37	Postgraduate	Rehabilitation Therapist	8	48
RN4	Female	33	College	Nurse	6	40
CG1	Female	51	Primary school	Caregiver	7	39
CG2	Female	46	Primary school	Caregiver	6	42
CG3	Female	45	Middle school	Caregiver	5	40
CG4	Female	46	Middle school	Caregiver	4	38
CG5	Male	53	Primary school	Caregiver	8	41
CG6	Female	45	College	Caregiver	8	45
CG7	Female	48	Primary school	Caregiver	8	39
CG8	Female	30	College	Caregiver	4	42
RT2	Male	39	College	Rehabilitation Therapist	12	46
RT3	Female	35	College	Rehabilitation Therapist	10	45
RN5	Female	38	College	Nurse	14	43
RN6	Female	30	College	Nurse	7	41
PR3	Male	35	Postgraduate	Rehabilitation physician	7	46

RN = Nurse; PR = Rehabilitation physician; RT = Rehabilitation Therapist; CG = Caregiver. Interview duration for stakeholders ranged from approximately 38 to 48 min.

**Table 4:** The profile of interviewed members of the general public.

Participants	Sex	Age	Education	Relationship	Interview duration (min)
GP1	Female	51	Middle school	Husband	46
GP2	Female	54	Primary school	Husband	43
GP3	Female	55	Primary school	Husband	47
GP4	Male	52	Primary school	Wife	40
GP5	Female	57	Middle school	Husband	45
GP6	Male	24	Postgraduate	Grandmother	50
GP7	Female	23	Postgraduate	Mother	40
GP8	Female	40	Postgraduate	Grandmother	44
GP9	Female	65	Primary school	Mother	44
GP10	Female	42	Postgraduate	Father	45
GP11	Female	36	Postgraduate	/	49
GP12	Male	49	College	/	39
GP13	Male	39	Postgraduate	/	43
GP14	Female	40	College	/	43
GP15	Female	52	College	/	45
GP16	Male	40	College	/	46
GP17	Male	29	College	/	41
GP18	Female	28	College	/	41
GP19	Female	43	College	Mother	40
GP20	Male	45	Postgraduate	/	42
GP21	Male	49	Middle school	/	45
GP22	Male	56	Middle school	/	46
GP23	Female	51	College	Husband	40
GP24	Female	54	Primary school	/	40

GP = General public in the communities. Interview duration for members of the general public ranged from approximately 39 to 50 min.

### 2.3 Data collection and analysis

Face-to-face interviews were conducted to facilitate rapport building and to allow the researcher to observe participants’ nonverbal expressions and reactions. Most interviews with older adults with stroke and their caregivers took place in nursing homes and community-based senior activity centers, while interviews with stakeholders and members of the general public were held in their workplaces or public meeting spaces. Interview duration varied by participant group, with interviews with older adults with stroke typically lasting around 30 min, and interviews with stakeholders and members of the general public lasting approximately 45 min. Detailed duration information is provided in Tables 2–4. Data saturation was reached when no new insights emerged regarding stigma or wheelchair-related attributes.

After completing the interviews, the analysis process commenced to systematically identify and interpret key themes. All interviews were audio-recorded, transcribed verbatim, and imported into ATLAS.ti 23 along with photographs of wheelchairs, to enable an integrated textual and visual analysis. A dedicated memo system was maintained within ATLAS.ti to document analytic decisions, reflections, and links between codes and raw data, forming a transparent audit trail. Transcripts were shared with participants for verification, and detailed notes were kept to support research transparency and traceability.

Thematic analysis followed Braun and Clarke’s (28) reflexive approach, emphasizing iterative engagement with data and researcher reflexivity. Coding employed both inductive and deductive strategies: deductive codes were derived from the conceptual framework, while inductive codes emerged organically from participants’ narratives. Theme development proceeded through three main stages: (1) initial coding and clustering of semantically related excerpts; (2) grouping of codes into candidate themes reflecting recurrent patterns of meaning; and (3) refinement and naming of final themes through collaborative review.

The first author conducted all interviews and took detailed field notes to ensure a deep understanding of the contextual meanings embedded in participants’ narratives. The corresponding author, who has extensive experience in qualitative research and aging-related design, participated in transcript review, coding validation, and theme refinement. Regular peer-debriefing sessions involving all seven authors were conducted. The first two authors collaborated to refine code clusters and preliminary themes, and the remaining five authors contributed by independently reviewing coded segments, triangulating across participant groups, and critically examining theme coherence and boundaries to enhance analytical rigor.

To enhance trustworthiness, several strategies were implemented. Member checking was performed by returning transcripts to participants for confirmation of accuracy. Peer debriefing between all the authors was carried out throughout the analysis to challenge interpretations and minimize individual bias. A detailed audit trail documenting analytical decisions and memos was maintained within ATLAS.ti. Triangulation was achieved by comparing perspectives across older adults with stroke, stakeholders, and members of the public, as well as between interview and observational data. In addition, reflexive memos were maintained throughout the research process to record the researcher’s assumptions and positionality, ensuring transparency in interpretation. Collectively, these strategies strengthened the credibility, dependability, and confirmability of the findings.

### 2.4 Ethics

This study was approved by the Ethics Committee of the researcher’s university (Reference No. JKEUPM-2023-632). All participants were informed about the study’s purpose, procedures, and potential implications, and written informed consent was obtained prior to participation to ensure voluntary involvement. Participants were assured of their right to withdraw from the study at any time without consequence, and that their identities and responses would remain strictly confidential. To protect participants’ privacy and data security, all interviews were conducted in private settings. Data were anonymized, and both audio recordings and transcripts were securely stored on password-protected devices accessible only to the research team. Special care was taken throughout the data collection process to minimize any potential distress and to ensure participants’ comfort, safety, and dignity.

## 3 Results

Themes related to the stigmatization of wheelchair use among older adults with stroke were identified through thematic analysis. A total of 30 initial codes were generated across all interview transcripts. These codes were subsequently organized into 12 code groups based on semantic similarity and conceptual relevance. Through iterative comparison, ongoing refinement, and collaborative discussions among the authors, these code groups were further synthesized into five overarching themes. The five final themes, namely symbolic impact on personal identity, perceived visual aesthetics, adaptability across daily living contexts, autonomy and control in wheelchair use, and ease of operation in wheelchair maneuvering, capture the core dimensions of stigma-related experiences identified in this study. A coding table illustrating the progression from initial codes to code groups, sub-themes, and final themes is provided in Appendix 2.

### 3.1 Symbolic impact on personal identity

All community respondents highlighted the wheelchair’s symbolic nature. Under this theme, three sub-themes emerged: the symbol of disability, the symbol of dependence, and the symbol of autonomy.

#### 3.1.1 Symbol of disability

In this subtheme, wheelchairs were recognized as markers of disability. This perspective was primarily expressed by relatives of older adults with stroke and other members of the general public. Four interviewees explicitly associated wheelchairs with mobility problems (CG6; GP4; GP9; GP10), arguing that they accentuated the disparity in mobility between older adults with stroke and others:

“…when purchasing a wheelchair from a store, it is typically grouped with medical equipment and not considered a common household item.” (GP4).

“… I feel that using a wheelchair amplifies the visible signs of disability and makes their physical limitations more obvious to me.” (GP10).

As the primary assistive device for older adults with stroke, the wheelchair was widely perceived as a visible marker of disability, particularly by members of the general public. Rather than being regarded as an ordinary mobility aid, it was frequently interpreted as a medical symbol that reinforced the distinction between older adults with stroke and the able-bodied. This reflects the social labeling dimension of the conceptual framework, where assistive devices amplify the visibility of impairment and act as entry points into broader processes of stigma and identity negotiation.

#### 3.1.2 Symbol of dependence

The wheelchair was also viewed as a symbol of dependence and burden. Respondents described both social and familial strains associated with its use. Two members of the general public noted that providing assistive facilities or escort services would demand additional social resources and financial support from the government (GP8, GP10). Three others emphasized the strain placed on families (GP2, GP5, GP23), arguing that wheelchair use could not restore independence and instead increased caregiving responsibilities for children and spouses.

“…They cannot rely on the young people for everything. They must adjust their mood after a stroke. The younger family members must go to work, and their children need to be sent to school, so it is best not to cause them trouble.” (GP9).

Some older adults with stroke also viewed themselves as a burden to their families after their stroke, as mentioned by older adults with stroke living in a nursing home:

“…Before, I could do many things, but now I cannot do anything except sit in a wheelchair. I would rather stay in a nursing home now; at home, I am stressed.” (SA7).

Thus, community respondents highlighted wheelchair use as a sign of social and familial dependence, while older adults with stroke internalized these expectations and often expressed feelings of guilt. This pattern illustrates how externally imposed meanings of wheelchairs as symbols of reliance are gradually absorbed by users, shaping their self-perception and reinforcing relational stigma within family dynamics.

#### 3.1.3 Symbol of autonomy

While more respondents mentioned the negative symbolism of wheelchairs, five community respondents saw wheelchairs as a symbol of the ability to live independently (GP1, GP2, GP4, GP6, GP8). Two of these interviewees felt that using a wheelchair for mobility could boost the self-confidence of older adults with stroke and enabled them to be active in their lives (GP2, GP4). Another respondent felt that using a wheelchair was a behavior to be encouraged:

“…Even though he has had a stroke, he is still able to move around freely, and he can still go to places where ordinary people go. Well, it is good enough to do things that ordinary people can do without the help of young people.” (GP6).

Three family members of older adults with stroke believed that their independence in using wheelchairs to accomplish what they could do before their stroke without seeking help from others demonstrated their autonomy (GP1, GP3, GP19). This more positive interpretation shows that wheelchair use can also serve as a site of identity negotiation, where individuals resist externally imposed labels of dependence by reframing mobility aids as tools of empowerment. In this sense, stigma is not static but fluid, contingent on both social context and individual agency.

Taken together, these three sub-themes reveal a layered process of social labeling. The initial label of disability is almost unavoidable once older adults with stroke adopt wheelchairs. From this starting point, symbolic interpretations diverge along two paths: wheelchairs as markers of dependence or as instruments of autonomy. Importantly, this divergence illustrates how wheelchair design and social framing actively shape the symbolic meaning attributed to older adults with stroke, influencing whether they are perceived by others or perceive themselves as dependent or empowered. Thus, identity symbolism surrounding wheelchairs should be understood as a dynamic field of negotiation between stigma and agency.

### 3.2 Perceived visual aesthetics

Five respondents discussed wheelchair aesthetics. They emphasized the lack of “design” in manual wheelchairs that were used by older adults with stroke and expressed their disappointment with the design, which they felt affected the image of these users (GP6, GP7, GP10, GP23, RN2). This theme contained two sub-themes, heaviness and coldness.

#### 3.2.1 Perceived visual heaviness

Three community respondents felt that manual wheelchairs gave a very bulky feeling and made it less easy to maneuver (GP6, GP7, GP23). They believed the bulky feeling of the wheelchair was mainly reflected in the materials and structure. One respondent reflected:

“…the exposed structural parts of the wheelchair felt cumbersome and created a visual burden. The structural parts of most wheelchairs are made of a bunch of tubular metal pipes spliced together, which makes them complicated and not lightweight.” (GP23).

The perception of “heaviness” extended beyond practical concerns of maneuverability to encompass broader symbolic meanings encoded in the material form. The visibility of exposed structures reinforced the medicalization of disability, framing the wheelchair less as a personal mobility aid and more as clinical apparatus. From a product semantics perspective, such visual coding aligns with stigma theory, wherein physical artefacts operate as stigma symbols that exaggerate bodily difference and constrain identity negotiation. In this sense, “heaviness” referred not only to physical mass but also to the cultural weight of a design language that encoded deficit and dependency. Negative aesthetic design further reinforced the separation from the able-bodied and diminished the possibility of alternative, empowering interpretations.

#### 3.2.2 Perceived emotional coldness

Four respondents talked about the wheelchairs’ icy feeling and sense of exclusion, which were mainly reflected in their color and material (GP7, GP10, GP23, RN2). One respondent reflected:

“…The manual wheelchairs are primarily grey and black, which makes people feel depressed. Meanwhile, the metal material of the wheelchair also gives people a cold feeling…” (GP10).

Two interviewees also compared manual wheelchairs and electric wheelchairs, and they thought that electric wheelchairs were more beautiful in both color matching and styling design (GP11, RN5).

The perception of “coldness” extended beyond color or tactile qualities to encompass deeper cultural meanings. Respondents described the dominance of grey and metallic materials as evoking detachment, reflecting aesthetics more closely associated with hospital environments than with domestic settings. This visual coding reinforced the medicalization of disability, positioning older adults with stroke within clinical rather than social environments. From a product semantics perspective, the use of cold, industrial materials communicated distance, otherness, and exclusion, rather than warmth, familiarity, or belonging. Such design choices amplified social distance and constrained opportunities for identity negotiation. In this sense, “coldness” was not merely an aesthetic property but a symbolic language that encoded disconnection, reinforcing the marginalization of wheelchair users and limiting the potential for positive re-signification.

Theme 2 directly extends Theme 1 by illustrating how the symbolic meanings of disability, dependence, and autonomy are materially encoded through aesthetic design. The “heaviness” and “coldness” of wheelchairs actively shape how symbolic labels are visually and culturally reinforced. In this way, aesthetics functions as the tangible medium through which identity symbolism is communicated, embedding social stigma into material form.

### 3.3 Adaptability across daily living contexts

There are three main themes related to adaptability: environmental adaptability, functional adaptability, and adaptability across rehabilitation stages. The interview results indicated that inadequate wheelchair adaptation can negatively impact the user’s mood, leading to feelings of frustration. Additionally, it can reinforce negative stereotypes about the user among bystanders.

#### 3.3.1 Environmental adaptability

Stroke limits the range of motion in older adults with stroke. One of the interviewed rehabilitation doctors mentioned that after having a stroke, many older adults are confined to their homes. However, he also talked about social activities that are critical to older people, and a prolonged lack of social activities can cause them to become depressed and frustrated, which affects their quality of life (PR2).

Two family members of older adults with stroke considered that the functions of the current wheelchairs were limited (GP4, GP9). They believed the current function of the wheelchairs restricted the user’s range of activities. They did not provide adequate assistance for older adults with stroke to carry out their social activities.

“…When the road surface is uneven, they cannot walk, and they cannot even push. They must rely on us to carry them across, which is too troublesome…” (GP4).

Two interviewees talked about the use of wheelchairs inside neighborhood hallways (GP7, GP14):

“…I often see various manual wheelchairs in stairwells, which occupy much space in our living areas. How can wheelchairs be used both at home and outdoors? That would be more convenient and would not bother other people.” (GP14).

Two other community interviewees felt that the current environment was not friendly to wheelchair users, such as the lack of signage and dedicated access on roads, a context that also places greater demands on wheelchair adaptability. Many older adults with stroke live in communities without elevators, making it very inconvenient for them to go up and down the stairs, and living in such communities also dramatically affects their social activities (GP12, GP20).

Limited adaptability across domestic and public environments does not simply create inconvenience but also reinforces social exclusion. Environmental and design incompatibility magnify users’ dependency on others, situating them as “outsiders” within everyday social spaces. In this sense, adaptability is not only a functional requirement but a symbolic marker of whether older adults with stroke are positioned as active participants or passive dependents in community life.

#### 3.3.2 Functional adaptability

If the wheelchair does not fulfil the user’s requirements, the wheelchair user will experience diminished self-confidence and feelings of dissatisfaction, which will impact the rehabilitation process. In addition to requiring a wheelchair for mobility, older adults with stroke also require assistance with eating, using the restroom, and storing their possessions. Using toileting needs as an illustration, numerous older adults with stroke require the aid of multiple caregivers during the act of toileting, which can often induce feelings of humiliation (CG1, CG4, CG7). A rehabilitation physician mentioned the problem of bed shifting in the early stages of stroke rehabilitation.

“…due to the patient’s weakness and limited lower limb strength, multiple nurses or family members are required to assist in moving the patient from the wheelchair to the bed. This process is embarrassing for the patient and cumbersome for the nurses and family members…” (PR2).

Poor adaptability to daily functional requirements encodes meanings of inadequacy and loss of dignity. A wheelchair that cannot support basic activities communicates a message of bodily insufficiency and dependency. This symbolic coding resonates with stigma theory, where lack of functional integration reinforces an identity of burden. Conversely, designs that accommodate multiple needs can enable re-signification, allowing the wheelchair to be seen as a facilitator of independence rather than a constant reminder of incapacity.

#### 3.3.3 Rehabilitation-stage adaptability

During various phases of rehabilitation, the wheelchair needs to cater to the specific rehabilitation requirements of the user. In the early stage of rehabilitation, for example, older adults with stroke typically require hospitalization for rehabilitation and treatment. In this context, wheelchairs play a crucial role in assisting the rehabilitation process for these patients. Two rehabilitation physicians have stated that during the initial rehabilitation phase, the wheelchair’s design must help older adults with stroke maintain a functioning posture (PR1, PR2). Alternatively, if the body tilts sideways, it might lead to safety issues that hinder the patient’s recovery and result in frustration:

“…the patient needs to maintain the functional position when using the wheelchair, and incorrect postures may lead to external rotation of the legs, which is not conducive to the patient’s rehabilitation.” (RT1).

A caregiver mentioned that in the later stages of rehabilitation, many older adults with stroke who live in the community or nursing homes need to rehabilitate independently and that a wheelchair may be able to play an assistive role in this process (CG8).

Adaptability across rehabilitation stages reflects the evolving needs of older adults with stroke. When wheelchairs fail to adjust to these shifting requirements, they not only frustrate users and caregivers but also symbolically trap older adults with stroke in a prolonged state of dependency. From a stigma perspective, such design limitations reinforce the image of immobility and helplessness, whereas wheelchairs that adapt across phases can embody progress, resilience, and recovery.

### 3.4 Autonomy and control in wheelchair use

Being able to maneuver a wheelchair independently was the most talked about topic among older adults with stroke who were interviewed, with participants repeatedly referring to difficulties related to motor control and wheelchair controllability. Four older adults with stroke shared their experiences with maneuvering wheelchairs, discussing how their inability to control the wheelchair has led to feelings of frustration and robbed them of the joys they once had in life (SA3, SA4, SA6, SA11). One older adult living in a nursing home expressed frustration when talking about life in a nursing home:

“… I cannot adjust my wheelchair on my own (sigh), and I must ask my caregiver to help me… (frustrated)” (SA6).

Two older adults with stroke experience stated that they were unable to push the wheelchair with their hands, so they chose to use their feet to move the wheelchair (SA4, SA10). An older adult with stroke living in a nursing home talked about his ambivalence about using a wheelchair:

“… With an electric wheelchair, I have concerns about not being able to control it. I feel that a manual wheelchair is safer, but I need the assistance of a caregiver every time, and I find it troublesome.” (SA1).

A rehabilitator suggested that wheelchair designs should focus on the users’ ability to care for themselves. It is essential for allowing older adults with stroke to live more independently with the assistance of their wheelchairs (RT1). The rehabilitator emphasized that enhancing wheelchair control and promoting self-care is crucial for helping these individuals return to everyday life and regain their confidence.

Several rehabilitation specialists and caregivers also raised concerns regarding wheelchair components that directly affect users’ autonomy and control, particularly armrests and footrests (CG3, CG5, RT3, PR2). Such design limitations further restrict users’ ability to maneuver the wheelchair independently. One caregiver explained that:

“…Older stroke patients often experience leg or foot stiffness, and existing footrests fail to meet their needs. Many seniors resort to removing the footrests…” (CG5).

Taken together, these accounts indicate that autonomy and control in wheelchair use constitute a central experiential dimension for older adults with stroke. Across interviews, difficulties in independent maneuvering were consistently associated with frustration, restricted mobility, and increased reliance on caregivers, whereas even limited forms of self-operation were described as meaningful sources of confidence and relief. These findings suggest that wheelchair controllability is closely intertwined with older adults’ everyday experiences of dependence and self-perception, positioning autonomy in wheelchair use as a salient factor in the stigmatization process.

### 3.5 Ease of operation in wheelchair maneuvering

From the perspectives of caregivers, nurses, and rehabilitation professionals, wheelchair operation was mainly discussed in terms of usability and ease of movement, reflecting concerns about operational burden in daily care contexts. In the early and middle stages of rehabilitation, in addition to family members, the leading wheelchair operators are caregivers and nurses. For caregivers in nursing homes and nurses in hospital rehabilitation departments, the ease of use of wheelchairs can impact their work efficiency. The weight and maneuverability of wheelchairs were the most criticized issues. Four respondents believed that the wheelchair is not light enough and lacks flexibility in operation (CG4, CG6, CG7, RN2).

“… Most of the caregivers in nursing homes were women, and when we encountered heavy older adults, we could not push them at all, and sometimes we could only drag the wheelchair along (anxiety).” (CG6).

Additional respondents highlighted the workload of daily wheelchair handling:

“After pushing wheelchairs back and forth all day, my arms and shoulders feel exhausted. There is no choice because the patients depend on us.” (CG2).

“When the wheelchair is stiff or hard to turn, I have to twist my body awkwardly. After a whole shift, my back feels sore.” (RN4).

Other interviewees talked about specific details of wheelchair operations. One rehabilitation physician mentioned the non-adjustable design of the wheelchair pedals, which occupied a significant amount of space and increased collisions with other things (PR1). A rehabilitation nurse expressed concerns that the bandages on the wheelchair were insufficient to completely immobilize the stroke victim, which could potentially endanger their safety when pushing the wheelchairs (RN3). Similarly, a caregiver expressed concerns about wheelchair brake problems, fearing they could jeopardize the safety of the person in the chair (CG3). These issues related to wheelchair maneuvering add to the stakeholders’ workload and are a source of negative emotions for them.

Across interviews, caregivers and healthcare professionals consistently emphasized ease of operation as a critical concern in everyday wheelchair use. Difficulties related to weight and maneuverability were repeatedly described as increasing physical strain, emotional stress, and negative caregiving experiences. These operational challenges were not perceived as isolated technical issues but as factors shaping daily care routines and interactions between caregivers and older adults with stroke, highlighting ease of operation as a key dimension influencing caregiving burden and relational dynamics.

Overall, the five identified themes collectively reveal a multilayered structure of stigma embedded in the wheelchair use experiences of older adults with stroke. From a stigma-mechanism perspective, Theme 1 (Symbolic Impact on Personal Identity) constitutes the core interpretive framework with the greatest explanatory power. It captures how the wheelchair operates as a social symbol that mediates identity negotiation between the self and others. The symbolic meanings attached to the wheelchair, including its representations of disability, dependence, and autonomy, provide the overarching framework that guides the interpretation of all subsequent themes.

In contrast, Themes 2–5 serve as supporting or explanatory sub-themes that demonstrate how symbolic meanings are materially and socially manifested through the design and use of wheelchairs. Together, these sub-themes construct a threefold logic of stigma encompassing the material, behavioral, and social dimensions of experience. Specifically, Theme 2 (Perceived Visual Aesthetics) and Theme 3 (Adaptability across Daily Living Contexts) correspond to the external or environmental layer, revealing how design form and contextual adaptability shape public perception and social visibility of wheelchair users. Meanwhile, Theme 4 (Autonomy and Control in Wheelchair Use) and Theme 5 (Ease of Operation in Wheelchair Maneuvering) pertain to the interactive or relational layer, emphasizing how usability, control, and caregiver interaction influence users’ sense of autonomy, dependency, and self-worth.

The interpretive significance of these themes follows an interconnected structure: while Identity Symbolism holds the most conceptual depth, it is continuously enacted and reinforced through the perceptual (visual), functional (behavioral), and relational (social) dimensions represented by the subsequent themes. In this way, stigma emerges not as a static perception but as a dynamic socio-symbolic process-one in which the visual expression, adaptive performance, and operational experience of the wheelchair collectively contribute to how users and others define its social meaning.

**Figure 2:**
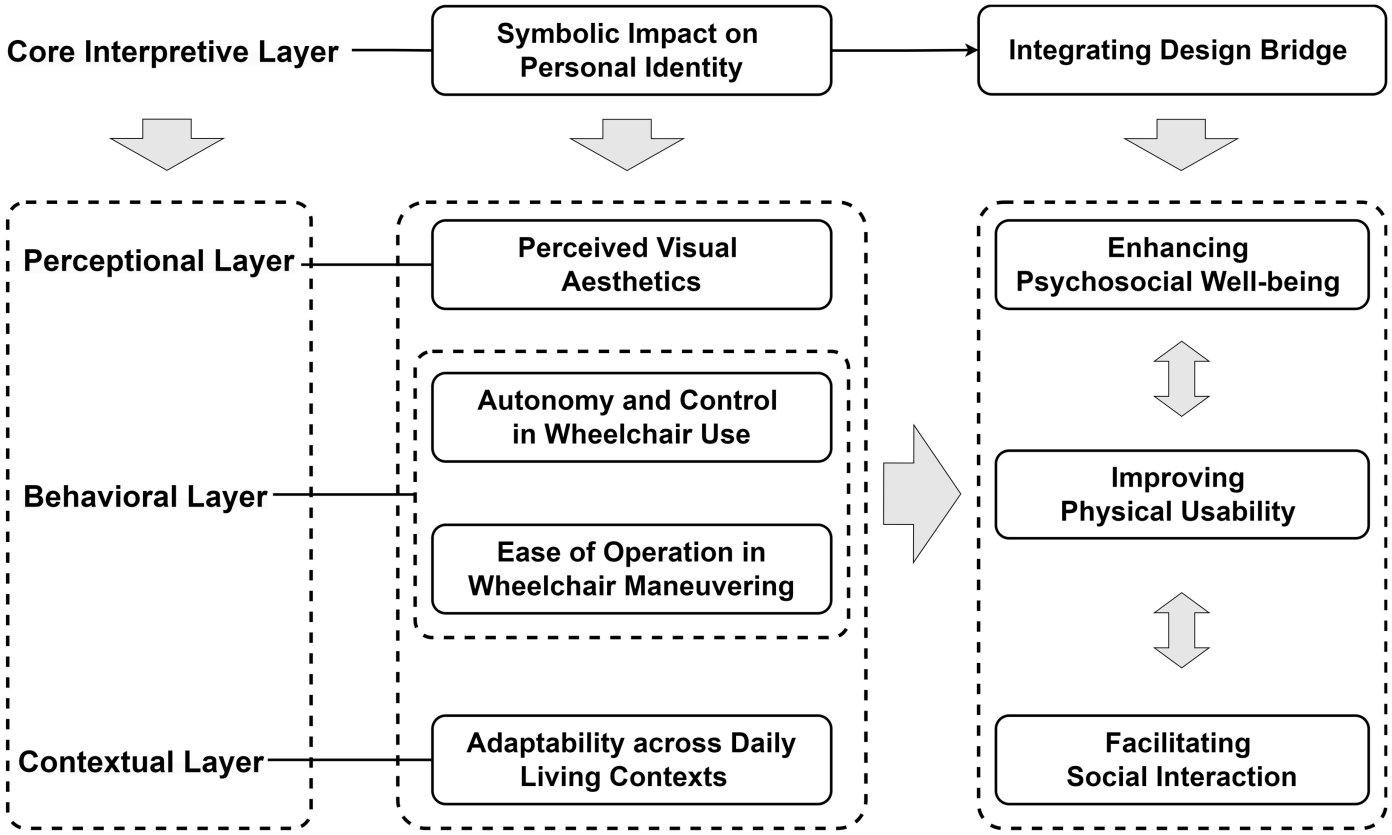
Design-oriented framework of wheelchair attributes influencing stigma among older adults with stroke.

This hierarchical yet intertwined structure underscores that wheelchair design attributes are not merely functional variables but mediators of social meaning, shaping both self-perception and social interaction. Consequently, addressing stigma through design requires an integrated approach that considers symbolic, material, and relational factors simultaneously, revealing how design can act as a bridge between physical usability and psychosocial well-being (see Figure 2).

## 4 Discussion

### 4.1 Wheelchair as a label

Stigma is a complex process of socialization (10). In this process, older adults with stroke are distinguished by physical disparities and a decline in their capacity to independently attend to their needs, leading society to categorize them as “disabled,” “dependent,” or “frail.” The labels influence their conduct and engagement with other social groups, leading to stereotypes and stigma (29). During the interviews, three community participants preferred creating spatial separation between themselves and older adults with stroke. Additionally, while expressing empathy for older adults with stroke, two nursing home caregivers often used discriminatory terms such as “paraplegic” and “crippled” to describe older adults with stroke during the interviews. For the members of the general public, perceptions of deterioration, mortality, and apprehension of limb deformity caused by a stroke evoke a combination of stereotypes (30). During this process of socialization, the wheelchair, which serves as a crucial necessity for the older adults with stroke, also assumes the role of a label and a marker of social standing for older adults with stroke.

Within the framework of labeling theory, the wheelchair serves the function of either strengthening or questioning the labels that are allocated to the older adults with stroke (31). The wheelchair promotes increased autonomy for older adults with stroke, potentially challenging the label of “disability” and “dependence,” thereby improving their self-perception (32). The inadequate empowerment of older adults with stroke through their wheelchairs may reinforce the stigma of “disability” and amplify the adverse effects linked to this label (3). The findings from the interviews indicate that wheelchairs can either contribute to stigmatization or, through innovative design and positive social cognitive shifts, act as a means to reduce stigmatization.

From the perspective of semiotics, a wheelchair serves as a “signifier,” physically representing a mobility tool, while as a “signified,” it may convey concepts such as “disability,” “dependence,” and “limited mobility” (33). The meaning of the wheelchair is not static but can be shifted based on societal perspectives, cultural origins, and individual encounters. Within the Chinese cultural context, older adults with stroke often internalize a strong sense of familial responsibility, deeply rooted in Confucian ethics and the moral expectation of contributing to family well-being (34). After experiencing a stroke, many older individuals perceive their physical decline as a failure to fulfil filial and familial duties, leading to feelings of guilt and self-stigma (35). This internalized sense of moral inadequacy frequently manifests in their attitudes toward assistive devices such as wheelchairs. For some, the use of a wheelchair signifies an unavoidable dependence that contradicts their lifelong role as family providers or caregivers, thereby intensifying their psychological burden and resistance to wheelchair use.

However, under the influence of contemporary rehabilitation discourse and policies such as Healthy China 2030, the meaning of the wheelchair is gradually shifting (36). Modern rehabilitation principles emphasize autonomy, functional recovery, and social participation, positioning the wheelchair not merely as a medical device symbolizing incapacity, but as an enabling tool that facilitates physical rehabilitation and enhances independent mobility. This transition reflects a broader cultural negotiation between traditional values of interdependence and modern ideals of independence.

For older adults with stroke, coping with this contradiction is both a psychological and a social process. Existing research indicates that AT is closely linked to users’ identity construction, while identity-related psychological factors shape the emotional and behavioral responses of older adults when using AT (37–39). This study expands research in this field by examining the link between wheelchair use and stigma among older adults with stroke, demonstrating that specific attributes of wheelchairs are directly associated with observable stigmatization processes among older adults with stroke in the Chinese context. By mapping wheelchair-related identity conflicts onto frameworks of health stigma and discrimination, this study elucidates how design features contribute to social labeling. Designs aimed at reducing stigma can begin with wheelchair attributes, integrating both symbolic and functional innovations. Culturally responsive wheelchair design not only supports individual well-being but also aligns with the objectives of long-term, primary-level stroke management, helping transform the symbolism of dependency into one of resilience, care, and continued integration within families and communities.

### 4.2 Differences in the perception of wheelchair aesthetics

In the interviews, most of the community respondents talked about their perceptions of the aesthetics of wheelchairs. However, older adults with stroke, their caregivers, and family members had little to say about the aesthetics of the wheelchair and were more concerned with its practicality. These differences stemmed from their different ways of experiencing the wheelchair and their different understandings of it as a symbol.

Stroke patients, as long-term users of wheelchairs, base their needs and perceptions of wheelchairs on the functions of use. Their aesthetic evaluation is more related to the experience of use. The same applies to caregivers and family members whose perception of wheelchairs is understood more through functional interaction. They are concerned with the maneuverability of the wheelchair. When asked about their aesthetic experience of the wheelchair, three family members indicated that simplicity and practicality were sufficient. Their aesthetic criteria were also more related to the functional experience.

Furthermore, the perceptions and emotional responses of wheelchair users are not static but evolve dynamically throughout the rehabilitation process. These changes are shaped by both functional recovery and emotional adaptation processes (56). For instance, as users regain partial mobility or achieve greater independence, they may increasingly attend to aesthetic aspects, integrating style, color, and design with functional considerations. Conversely, during the early stages of rehabilitation, users typically prioritize safety, comfort, and ease of use over appearance.

Bystanders’ experience is primarily visual and based on their observation of the wheelchair and its user, although some also have experience with wheelchairs. They are more likely to judge the wheelchair’s aesthetics based on color, design style, innovativeness, etcetera (40). Besides, for bystanders, the aesthetic value of a wheelchair also encompasses social recognition. A wheelchair is not only a physical tool but also a social symbol. The aesthetics of a wheelchair are regarded as an extension of the user’s identity, which influences bystanders to view and evaluate the user’s social status and quality of life (41).

From the social identity theory perspective, the aesthetic differences between older adults with stroke and the general public also stem from their sense of belonging to different social groups. According to Berger and Heath (42), individuals often use products and their aesthetic attributes as identity signals to align with their social group while distinguishing themselves from others. The process shapes aesthetic standards and reinforces group boundaries, which can influence perceptions that are related to assistive devices such as wheelchairs. Older adults with stroke often categorize themselves as wheelchair users or mobility-restricted groups, and they respond to identity threats by choosing designs that better express their status and value in the face of adverse social perceptions of ageing and stroke (10). Therefore, their focus on wheelchairs is more on functionality, comfort, and convenience (2).

In contrast, empirical research indicates that social factors such as peer conformity pressures and perceived social evaluation significantly shape aesthetic judgments, showing that preferences for visual features are socially embedded rather than purely individual (61). In addition, studies on assistive technologies demonstrate that the aesthetic appeal and perceived social acceptability of device designs are closely linked to stigma, suggesting that aesthetic features operate as social signals that influence both user acceptance and public perception (62). Collectively, these findings indicate that members of the general public’ evaluations of assistive product appearance are shaped by prevailing social norms and shared cultural aesthetic standards. Within this context, dominant mainstream aesthetic expectations guide community respondents’ perceptions of wheelchair aesthetics.

Overall, the findings highlight aesthetic differences among various groups regarding wheelchairs and emphasize the dynamic nature of aesthetic preferences among wheelchair users. These discoveries offer crucial design insights: designers should balance functionality and visual appeal when developing wheelchairs, ensuring the equipment not only meets operational and comfort requirements but also conveys positive social and psychological significance. Aesthetically pleasing and practical designs can reduce potential discrimination; incorporating adaptive design features allows adjustments as users progress through rehabilitation stages and changing daily needs, thereby better supporting their evolving user experience and social participation.

### 4.3 Multiple identities of caregivers

Caregivers of older adults with stroke (including family members and institutional nursing staff) often inhabit multiple and overlapping identities, and this diversity shapes their perceptions, attitudes, and emotional responses toward both older adults with stroke and wheelchair use. On one level, their “bound identity” as emotionally attached actors reflects relationships formed through kinship and long-term caregiving, which nurture profound concern for the older adults’ physical and psychological well-being (43). They empathize with the users’ struggles and actively seek to protect their dignity and autonomy, especially when witnessing or anticipating negative societal attitudes. This bound identity is characterized by moral commitment, affective labor, and a strong sense of responsibility, resonating with Confucian ideals of care and filial obligation that structure long-term stroke management within Chinese families (44).

However, this emotional investment also coexists with strain. Caregiving fatigue, emotional exhaustion, and moral distress may emerge when the demands of caregiving conflict with the caregiver’s own well-being (43). The difficulties and frustrations of caregiving can create ambivalent emotions, combining empathy with frustration, and may consequently strengthen the internalization of social stigma (45). In the context of long-term stroke rehabilitation, attention should also be directed toward the emotional and psychosocial well-being of caregivers. They ought to be regarded not merely as providers of assistance, but as individuals who themselves need continuous emotional support, psychological resilience, and empowerment.

In long-term stroke rehabilitation, the operational characteristics of wheelchairs play a pivotal role in shaping caregivers’ physical experiences, emotional responses, and stigma-related perceptions. When wheelchairs are heavy, rigid, or poorly designed, they significantly increase physical strain and emotional stress for caregivers, many of whom are women responsible for handling older adults with limited mobility. Excessive push-pull forces, awkward postures, and repetitive maneuvering tasks have been shown to elevate the physical burden placed on caregivers (46). From a stigma perspective, these operational difficulties extend beyond technical inconvenience. They contribute to what has been described as courtesy stigma-the stigma experienced by individuals associated with stigmatized persons. Caregivers who struggle with unwieldy wheelchairs may begin to perceive themselves as being weighed down by care tasks, which can reinforce negative attitudes toward older adults with stroke (43, 47). At the same time, older adults who observe visible frustration, discomfort, or anxiety in their caregivers may internalize feelings of guilt and shame about “being a burden,” thereby compounding their own self-stigma.

Empirical research further demonstrates that frequent toileting assistance and wheelchair transfer tasks are significantly associated with shoulder pain, upper back discomfort, and wrist-hand strain among caregivers (48). Operational ease is thus more than a matter of efficiency; it symbolically shapes the relational dynamics between caregivers and wheelchair users. Designs that minimize physical strain, optimize handle ergonomics, reduce rolling resistance, and improve maneuverability can reduce caregivers’ workload, alleviate emotional frustration, and thereby weaken the circulation of courtesy stigma. By contrast, poorly designed wheelchairs amplify perceptions of dependency and inconvenience, positioning older adults with stroke as sources of burden within care relationships.

At the same time, caregivers also occupy a “bystander identity” as social participants influenced by mainstream aesthetic and health ideals. In this role, they are sensitive to the visual image and symbolic meanings of wheelchairs, often perceiving them as reflections of both user identity and care quality (26). Their aesthetic evaluations, shaped by broader cultural norms, coexist with their practical concerns, generating tensions between functional pragmatism and social desirability.

Overall, caregivers function both as emotionally invested agents and socially influenced observers-a dual positioning that creates a complex arena for negotiating ethical responsibilities and symbolic meanings. Their role embodies both care and control, and encompasses empathy alongside exhaustion. These contradictions underscore the need for holistic, ethically grounded design strategies that not only address users’ rehabilitation needs but also support caregivers’ emotional labor within the long-term care ecosystem. Adopting such an approach promotes a more humane and sustainable model of stroke care.

Building on this understanding, several design implications emerge. First, caregivers’ functional burdens highlight the importance of enhancing controllability features-such as lightweight frames, effort-saving propulsion mechanisms, adjustable push handles, and more intuitive braking controls. Improving these features can significantly reduce physical strain, enhance operational confidence, and reinforce caregivers’ perceived competence and professional fulfillment. Second, because caregivers’ “bystander identity” is shaped by mainstream aesthetic norms, wheelchair design must address visual and symbolic concerns as well. This includes creating aesthetically harmonious forms, using warmer and less clinical material palettes, minimizing overtly medical features, and incorporating design cues that convey dignity rather than disability. Such approaches help reduce the symbolic stigma associated with assistive devices, supporting caregivers’ desire to present the care recipient in a socially acceptable and affirming manner. Wheelchairs designed with these considerations in mind not only ease caregiving tasks but also contribute to relational well-being, empathy, and mutual dignity between caregivers and older adults with stroke-advancing a more integrated model of long-term, community-based stroke management.

### 4.4 Self-efficacy in older adults with stroke

Mobility is critical for older people after a stroke as it directly impacts their quality of life, social participation, self-esteem, and emotional well-being, as well as maintaining their sense of identity and belonging (49). When older people can maneuver their wheelchairs independently, this ability reinforces their confidence that they can take control of their daily lives. Being able to maneuver their wheelchairs independently will help them maintain their dignity and reduce the stigma that is associated with being dependent on others (32). The loss of independence means that they need to rely on the assistance of others to complete their daily activities, such as washing, dressing, and toileting, which will directly affect their quality of life (63). Furthermore, a stroke can limit an older person’s participation in social activities. Reduced socialization opportunities due to mobility problems can lead to social isolation and emotional loneliness (50).

For older adults with stroke, the ability to maneuver a wheelchair independently represents more than a matter of physical mobility. Mobility assistive devices function as extensions of the user’s body and autonomy, and their usability directly influences users’ sense of control and psychological well-being (51). From this perspective, wheelchair use becomes a critical site where self-stigma is either reinforced or alleviated. When wheelchair design is poorly aligned with users’ physical capabilities and cognitive expectations, it can generate frustration and reduce the willingness of users to engage with assistive technologies (52). Inability to operate the wheelchair independently often leads not only to frustration but also to an internalized sense of incapacity, reinforcing fears of being burdensome and dependent. Moreover, anxiety toward technology has been shown to reduce older adults’ willingness to adopt rehabilitation assistive devices (53).

Conversely, human-centred AT design has been shown to enhance perceived usability, technological comprehensibility, and informational clarity, all of which are key sources of self-efficacy (54). When wheelchairs facilitate even partial autonomy, they create opportunities for older adults with stroke to reclaim agency. Independence in maneuverability supports self-efficacy, enabling individuals to renegotiate their social identity not solely as patients, but as capable participants in everyday life. From the perspective of stigma, such autonomy mitigates self-stigma by disrupting the internalization of dependency and by symbolically affirming competence and dignity.

From a design perspective, the wheelchair can play an instrumental role in sustaining long-term recovery and everyday well-being after stroke. Design attributes that enhance controllability, intuitive operation, and adaptive mobility help users rebuild confidence in movement and reinforce a sense of mastery in their surroundings. At the same time, aesthetic and symbolic qualities, including a lightweight, personalized, and lifestyle-oriented appearance, can reshape how users and others perceive the device, transforming it from a sign of dependency into an emblem of self-management and resilience. Enhancing self-efficacy through wheelchair design therefore requires attention to both functional empowerment, which is achieved through flexibility, precision, and comfort, and symbolic empowerment, which is expressed through aesthetics that affirm dignity and agency. When these two dimensions are effectively integrated, the wheelchair supports not only physical mobility but also emotional stability, social connectedness, and continuity of self in everyday contexts. Together, these outcomes are essential to sustained recovery and holistic care for older adults with stroke.

Taken together, the findings of this study respond to the proposed conceptual framework of stigmatization, which conceptualizes stigma as a dynamic socio-symbolic process shaped through interactions among older adults with stroke, caregivers, and bystanders. The interview data reveal that each group occupies a distinct yet interdependent position within the stigmatization cycle. Older adults with stroke are frequently labeled as “disabled” or “burdensome” and face a heightened risk of internalizing negative social judgements. As the direct targets of stigma, they are often perceived as “weak” or “dependent” due to the intersection of ageing and physical impairment. Caregivers, meanwhile, experience associative stigma as they navigate their dual identities as emotionally invested actors and socially influenced observers, balancing care responsibilities with external expectations. Community bystanders further contribute to the symbolic construction of stigma through implicit aesthetic norms and moral assumptions surrounding disability and assistive device use. Importantly, these roles are not fixed but evolve through everyday interactions. Within this dynamic process, the wheelchair functions simultaneously as a material tool and a symbolic artefact. The findings demonstrate that specific design attributes of wheelchairs can actively mediate these interactions, either reinforcing stigma or helping to disrupt it.

The five identified themes reveal how design meaningfully interacts with social and psychological mechanisms of stigma. “Symbolic impact on personal identity” serves as the interpretive core that frames how wheelchairs are perceived either as signs of disability or as tools of agency. “Perceived visual aesthetics” and “adaptability across daily living contexts” correspond to the perceptional layer and contextual layer, shaping social perception and influencing the integration of wheelchairs into everyday contexts. “Autonomy and control in wheelchair use” and “ease of operation in wheelchair maneuvering” define the behavioral layer, directly influencing users’ sense of autonomy, confidence, and harmony in caregiver relationships. Collectively, these dimensions form an interdependent structure in which symbolic, functional, and relational factors jointly determine the emergence of stigma and the potential for empowerment.

From a design translation perspective, prioritizing control capability and identity symbolism provides the potential for practical, real-world impact. Enhancing control capability through lightweight structures, intuitive interfaces, or single-hand operation can strengthen users’ perceived agency and confidence in long-term use. At the same time, redefining the symbolic identity of wheelchairs through design languages that reflect everyday lifestyles rather than clinical aesthetics can help normalize their presence in social environments and mitigate public prejudice. Ultimately, effective wheelchair design must integrate symbolic insight, material innovation, and social empathy to transform the wheelchair from a passive medical device into an active medium of empowerment and social connection. In doing so, design contributes not only to physical usability but also to psychosocial well-being and social interaction. Together, these dimensions form the foundation for the long-term well-being of older adults with stroke (see Table 5).

**Table 5:** Summary of key insights and contributions.

Analytical dimension/theme	Key findings from this study	Design implications
Symbolic impact on personal identity	This study highlights the relational and symbolic mediation of stigma through wheelchair design. Wheelchairs are interpreted not only as mobility aids but as symbolic markers of disability, dependence, or dignity. Identity meanings are negotiated among older adults with stroke, caregivers, and bystanders.	Reframing wheelchair identity through non-medicalized design language, everyday-product aesthetics, and lifestyle-oriented form can support dignity and agency.
Perceived visual aesthetics	This study extends existing research by situating aesthetics within caregiver-bystander-user dynamics and social signaling. Aesthetic simplicity and reduced medical cues help caregivers and users avoid unwanted social attention and mitigate public stigma.	Use warm materials, harmonious proportions, and visually normalized forms to reduce stigma without compromising functionality.
Adaptability across daily living contexts	This study links adaptability to psychosocial stigma reduction and relational well-being. Limited adaptability across contexts (home, community, care facilities) reinforces dependency and stigma for both users and caregivers.	Design modular, context-responsive wheelchairs that adapt to daily life scenarios and changing rehabilitation needs.
Autonomy and control in wheelchair use	This study highlights control capability as a stigma-sensitive design attribute with relational consequences. Loss of control is a major trigger for self-stigma among older adults with stroke.	Prioritize lightweight structures, intuitive interfaces, and single-hand operation to enhance agency and confidence.
Ease of operation in wheelchair maneuvering	This study frames ease of operation as a mediator of associative stigma. Operational difficulty increases caregiver burden and reinforces perceptions of dependency and incompetence.	Optimize braking systems, propulsion efficiency, and caregiver-facing controls to reduce physical and emotional strain.
Stigma process	This study conceptualizes stigma as a dynamic socio-symbolic process mediated by design. Stigma emerges through dynamic interactions among older adults with stroke, caregivers, bystanders, and the wheelchair as a material-symbolic artefact.	Treat wheelchair design as an active mediator shaping social interaction, not merely a functional aid.
Conceptual contribution	It integrates Health Stigma and Discrimination Framework with design research to explain how material artefacts mediate stigma.	The framework provides a design-oriented interpretation of stigma theory applicable to assistive product development.

## 5 Conclusion

This study examined wheelchair-related stigma among older adults with stroke and highlighted the interconnected roles of older adults with stroke, caregivers, and bystanders in the stigmatization process. The findings demonstrate that stigma extends beyond an individual psychological experience and operates as a dynamic relational process, in which material artefacts such as the wheelchair play a mediating role. By integrating qualitative interviews with a design-oriented analytical framework, this study shows that wheelchair design simultaneously shapes physical usability, social perception, and emotional well-being.

Five interrelated design themes-namely, symbolic impact on personal identity, perceived visual aesthetics, adaptability across daily living contexts, autonomy and control in wheelchair use, and ease of operation in wheelchair maneuvering-were identified as key mechanisms through which stigma is either reinforced or mitigated. These design attributes affect users’ sense of agency and social positioning, while also shaping caregivers’ emotional burden and public interpretations of disability. These findings extend existing stigma research by demonstrating how design attributes operate across symbolic, functional, and relational dimensions, positioning the wheelchair as both a material tool and a social mediator.

By translating stigma-related insights into concrete design implications, this study contributes a design-oriented perspective to gerontechnology and stroke rehabilitation research. Thereby, it advances an evidence-based understanding of how assistive device design can move beyond clinical functionality to actively support dignity, autonomy, and relational well-being in long-term stroke care.

### 5.1 Limitations and future studies

This study has several limitations. First, the sample size of the study was limited, and all the participants resided in urban areas, excluding older adults with stroke who lived in rural regions. The socio-cultural context, medical resources, and social support in rural locations may differ from those in urban environments, affecting the stigma experience. Secondly, the participants in this study utilized manual wheelchairs. The distinct technical characteristics and usage contexts of electric wheelchairs compared to manual wheelchairs necessitate a closer examination of how design attributes influence the stigma that is associated with them. Moreover, the study did not conduct comparative analyses to examine how characteristics such as age and gender might have influenced participants’ perceptions of stigma and their design preferences.

Future research could therefore adopt comparative and participatory approaches to extend these findings. Subgroup analyses may further explore how factors such as gender, age, education, and living environments shape attitudes toward wheelchair use and perceptions of stigma, providing a more nuanced understanding of these experiences. Cross-context studies comparing urban and rural populations could also highlight how environmental and cultural variables influence the formation and expression of stigma. Building directly on the present findings, subsequent studies should examine how specific stigma-related design attributes operate across user groups and care contexts over time. Longitudinal or co-design-based investigations could assess how sustained engagement with stigma-sensitive wheelchair designs influences users’ self-perception, caregivers’ emotional burden, and patterns of social interaction, thereby clarifying the long-term psychosocial impact of design interventions.

## Data Availability

The original contributions presented in the study are included in the article/Supplementary material, further inquiries can be directed to the corresponding author.

## References

[1] Yang W Wu B Tan SY Li B Lou VWQ Chen ZA . Understanding health and social challenges for aging and long-term care in China. Res Aging. (2021) 43:127–35. doi: 10.1177/0164027520938764, 32677535 PMC7961665

[2] Barker DJ Reid D Cott C. Acceptance and meanings of wheelchair use in senior stroke survivors. Am J Occup Ther. (2004) 58:221–30. doi: 10.5014/ajot.58.2.221, 15068158

[3] Barbareschi G Carew MT Johnson EA Kopi N Holloway C. “When they see a wheelchair, they've not even seen me”: factors shaping the experience of disability stigma and discrimination in Kenya. Int J Environ Res Public Health. (2021) 18:4272. doi: 10.3390/ijerph18084272, 33920601 PMC8073617

[4] Jia J Ning Y Chen M Wang S Li Y Yang H. Ending age discrimination and stigma to promote healthy ageing in China. Lancet. (2022) 400:1907–9. doi: 10.1016/S0140-6736(22)02362-5, 36423649

[5] Lu Q Wang D Fu L Wang X Li L Jiang L . The effect of stigma on social participation in community-dwelling Chinese patients with stroke sequelae: a cross-sectional study. Clin Rehabil. (2022) 36:407–14. doi: 10.1177/02692155211050558, 34787017

[6] Zhu M Zhou H Zhang W Deng Y Wang X Zhang X . Stigma experienced by Chinese patients with stroke during inpatient rehabilitation and its correlated factors: a cross-sectional study. Top Stroke Rehabil. (2019) 26:342–8. doi: 10.1080/10749357.2019.1605759, 31104577

[7] Goffman E. Stigma: Notes on the Management of Spoiled Identity. New York: Simon and Schuster (2009).

[8] Scambler G. Health-related stigma. Sociol Health Illn. (2009) 31:441–55. doi: 10.1111/j.1467-9566.2009.01161.x, 19366430

[9] Corrigan PW Bink AB Schmidt A Jones N Rüsch N. What is the impact of self-stigma? Loss of self-respect and the ‘why try’ effect. J Ment Health. (2016) 25:10–5. doi: 10.3109/09638237.2015.1021902, 26193430

[10] Major B O’Brien LT. The social psychology of stigma. Annu Rev Psychol. (2005) 56:393–421. doi: 10.1146/annurev.psych.56.091103.070137, 15709941

[11] Li C Hu M Yang T Shao X Zheng D. Correlates of stigma for poststroke patients: a meta-analysis. J Clin Nurs. (2023) 32:1952–62. doi: 10.1111/jocn.16250, 35181955

[12] Ribeiro-Gonçalves JA Costa PA Leal I. Loneliness, ageism, and mental health: the buffering role of resilience in seniors. Int J Clin Health Psychol. (2023) 23:100339. doi: 10.1016/j.ijchp.2022.100339, 36168598 PMC9485034

[13] Stangl AL Earnshaw VA Logie CH Van Brakel WC Barré I Dovidio JF . The health stigma and discrimination framework: a global, crosscutting framework to inform research, intervention development, and policy on health-related stigmas. BMC Med. (2019) 17:1–13. doi: 10.1186/s12916-019-1271-3 30764826 PMC6376797

[14] Dong X. Elder rights in China: care for your parents or suffer public shaming and desecrate your credit scores. JAMA Intern Med. (2016) 176:1429–30. doi: 10.1001/jamainternmed.2016.5011, 27571539 PMC7422934

[15] Sun T Zhang S-E Yan M Lian T Yu Y Yin H . Association between self-perceived stigma and quality of life among urban Chinese older adults: the moderating role of attitude toward own aging and traditionality. Front Public Health. (2022) 10. doi: 10.3389/fpubh.2022.767255, 35223724 PMC8873104

[16] Carneiro L Rebelo F Noriega P Faria Pais J. Could the design features of a wheelchair influence the user experience and stigmatisation perceptions of the users? In: Editor AA Editor BB Editor CC, editors. Advances in ergonomics in design: Proceedings of the AHFE 2017 international conference on ergonomics in design: Springer International Publishing (2018). 841–50.

[17] Gaffney C. An exploration of the stigma associated with the use of assistive devices. J Sociol. (2010) 3:67–78.

[18] Davis A McMahon CM Pichora-Fuller KM Russ S Lin F Olusanya BO . Aging and hearing health: the life-course approach. Gerontologist. (2016) 56:S256–67. doi: 10.1093/geront/gnw033, 26994265 PMC6283365

[19] Park H Kim JH. The use of assistive devices and social engagement among older adults: heterogeneity by type of social engagement and gender. GeroScience. (2023) 46:1385–94. doi: 10.1007/s11357-023-00910-6, 37581756 PMC10828457

[20] Krippendorff K. The semantic turn: A new Foundation for Design. Boca Raton, FL: CRC Press (2005).

[21] Kumar M Noble CH. Beyond form and function: why do consumers value product design? J Bus Res. (2016) 69:613–20. doi: 10.1016/j.jbusres.2015.05.017

[22] Vaes K. 2019. Design for empowerment, the stigma-free design toolkit. In proceedings of the 20th congress of the international ergonomics association (IEA 2018), 824, 1012–1030

[23] Schröppel T Miehling J Wartzack S. The role of product development in the battle against product-related stigma: a literature review. J Eng Des. (2021) 32:247–70. doi: 10.1080/09544828.2021.1879031

[24] Shinohara K Wobbrock J. Self-conscious or self-confident? A diary study conceptualizing the social accessibility of assistive technology. ACM Trans Access Comput. (2016) 8:1–31. doi: 10.1145/2827857

[25] Kumari N Lenka U. Employment and retention of differently-abled people in the workplace through assistive technologies. Int J Digit Technol. (2023) 2

[26] dos Santos AD Ferrari AL Medola FO Sandnes FE. Aesthetics and the perceived stigma of assistive technology for visual impairment. Disabil Rehabil Assist Technol. (2022) 17:152–8. doi: 10.1080/17483107.2020.1768308, 32501732

[27] Major B Dovidio JF Link BG. The Oxford handbook of stigma, discrimination, and health. Oxford: Oxford University Press (2018).

[28] Braun V Clarke V. Using thematic analysis in psychology. Qual Res Psychol. (2006) 3:77–101. doi: 10.1191/1478088706qp063oa

[29] Link BG Phelan JC. Stigma power. Soc Sci Med. (2014) 103:24–32. doi: 10.1016/j.socscimed.2013.07.035, 24507908 PMC4451051

[30] O’Connor ML McFadden SH. A terror management perspective on young adults’ ageism and attitudes toward dementia. Educ Gerontol. (2012) 38:627–43. doi: 10.1080/03601277.2012.695016

[31] Link BG Phelan JC. Conceptualizing stigma. Annu Rev Sociol. (2001) 27:363–85. doi: 10.1146/annurev.soc.27.1.363

[32] Chu Q Ramli SH Ahmad SAB Mansor NB Rokhani FZB Li Z . Empowering post-stroke older adults through wheelchair development: a conceptual synthesis for stigma reduction and well-being enhancement. Disabil Rehabil Assist Technol. (2024):1–13. doi: 10.1080/17483107.2024.2424878 39504232

[33] Pedersen H Søderstrøm S Kermit PS. Assistive activity technology as symbolic expressions of the self. Technol Disabil. (2019) 31:129–40. doi: 10.3233/TAD-190236

[34] Zhang L Han Y Ma Y Xu Z Fang Y. Eastern perspectives on roles, responsibilities and filial piety: a case study. Nurs Ethics. (2021) 28:327–45. doi: 10.1177/0969733020934143, 32666888

[35] Pan Y Chen R Yang D. The relationship between filial piety and caregiver burden among adult children: a systematic review and meta-analysis. Geriatr Nurs. (2022) 43:113–23. doi: 10.1016/j.gerinurse.2021.10.024, 34864295

[36] Li B Ma X Yu Y Wang G Zhuang N Liu H . “Healthy China 2030”: promoting health and longevity of the whole nation In: Tutorial for outline of the healthy China 2030 plan. Singapore: Springer Singapore (2020). 1–9.

[37] McNicholl A Desmond D Gallagher P. Feeling valued: the interplay of assistive technology and identity. Disabil Rehabil Assist Technol. (2024) 19:2580–91. doi: 10.1080/17483107.2023.2294987, 38116935

[38] Morejon Hernandez Y Moyo GS Desmond D. Identity concerns and older people’s use of assistive technology: a scoping review. Assist Technol. (2025):1–11. doi: 10.1080/10400435.2025.2591838, 41359879

[39] Rasoulivalajoozi M Cucuzzella C Farhoudi M. Domains of wheelchair users’ socio-emotional experiences: design insights from a scoping review. Disabil Health J. (2025) 18:101829. doi: 10.1016/j.dhjo.2025.101829, 40164524

[40] Hekkert P Leder H. Product aesthetics In: Product Experience: Elsevier (2008). 259–85.

[41] Lindström M Bäckström A-C Henje C Stenberg G. “When I use the electric wheelchair, I can be myself”: real-life stories about occupational identity construction. Scand J Occup Ther. (2022) 30:1368–82. doi: 10.1080/11038128.2022.2093268, 35786150

[42] Berger J Heath C. Where consumers diverge from others: identity signaling and product domains. J Consum Res. (2007) 34:121–34. doi: 10.1086/519142

[43] Lu Q Mårtensson J Zhao Y Johansson L. Living on the edge: family caregivers’ experiences of caring for post-stroke family members in China: a qualitative study. Int J Nurs Stud. (2019) 94:1–8. doi: 10.1016/j.ijnurstu.2019.02.016, 30928717

[44] Han K Chen Y Li M Cui L. Using a mixed-method approach to explore the factors influencing the family resilience of stroke survivors in China. J Multidiscip Healthc. (2024) 17:275–87. doi: 10.2147/JMDH.S439737, 38264410 PMC10804964

[45] Su JA Chang CC. Association between family caregiver burden and affiliate stigma in the families of people with dementia. Int J Environ Res Public Health. (2020) 17:2772. doi: 10.3390/ijerph17082772, 32316454 PMC7215659

[46] Kadomatsu H. Sudo S. Nakashima Y. Yamamoto M. 2019. Physical burden on caregivers pushing wheelchairs, in proceedings of the 2019 IEEE/SICE international symposium on system integration (SII). IEEE.

[47] Zhang J Lee DTF. Meaning in stroke family caregiving in China: a phenomenological study. J Fam Nurs. (2019) 25:260–86. doi: 10.1177/1074840719841359, 30994394

[48] Hsieh PL Lee YC Yang SY Lin YL Huang YR. Association between work content and musculoskeletal disorders among home caregivers: a cross-section study. Ind Health. (2021) 60:514–24. doi: 10.2486/indhealth.2021-0160, 34819407 PMC9726607

[49] Uchenwoke CI Arinze BO Nwankwo MJ Umunnah JO. Quality of life, self-esteem, self-efficacy, and social participation of persons living with mobility-related disability using mobility aids devices within select Nigerian communities. Disabil Rehabil Assist Technol. (2023) 18:532–7. doi: 10.1080/17483107.2021.1881173, 33555947

[50] Kam JE Choo PL. Feeling lonely and dissatisfied: understanding social network functioning in stroke survivors. BMC Psychol. (2024) 12:558. doi: 10.1186/s40359-024-01986-1, 39407283 PMC11481469

[51] Joskow R Patel D Landre A Mattick K Holloway C Danemayer J . Understanding the impact of assistive technology on users’ lives in England: a capability approach. Bioengineering. (2025) 12:750. doi: 10.3390/bioengineering12070750, 40722442 PMC12292795

[52] Morris L Cramp M Turton A. User perspectives on the future of mobility assistive devices: understanding users’ assistive device experiences and needs. J Rehabil Assist Technol Eng. (2022) 9:20556683221114790. doi: 10.1177/20556683221114790, 35983071 PMC9380214

[53] Goodarzi F Barati M Rahbar S Ayubi E Cheraghi P Bashirian S. Factors associated with intention to use rehabilitation assistive technologies in older adults. Disabil Rehabil Assist Technol. (2025):1–12. doi: 10.1080/17483107.2025.2579038 41182047

[54] Al-Tamimi A-K Hewitt L Cameron D Salem M Moemeni A. Challenges of integrating assistive technologies and robots with embodied intelligence in the homes of older people living with frailty. Appl Sci. (2025) 15:8415. doi: 10.3390/app15158415

[55] Pescosolido BA Martin JK. The stigma complex. Annu Rev Sociol. (2015) 41:87–116. doi: 10.1146/annurev-soc-071312-145702, 26855471 PMC4737963

[56] Rasoulivalajoozi M Cucuzzella C Farhoudi M. The dynamics of affective experiences with wheelchair use during rehabilitation: a qualitative study through physiotherapists' perspectives. Acta Psychol. (2025) 256:105022. doi: 10.1016/j.actpsy.2025.105022, 40233653

[57] Yin RK. Case study research and applications: Design and methods. 6th ed. Thousand Oaks, CA: Sage Publications (2018).

[58] Zhao YX Hua X Ren M Ouyang C Chen Y Li X Yin et al. Increasing Burden of Stroke in China: A Systematic Review and Meta-Analysis of Prevalence, Incidence, Mortality, and Case Fatality. Intern J Stroke: Official J Intern Stroke Soc. (2023) 18:259–267. doi: 10.1177/17474930221135983 36274585

[59] Etikan I Musa S. A. Alkassim RS. Comparison of Convenience Sampling and Purposive Sampling. American J Theor Appli Statis. (2016) 5:1–4. doi: 10.11648/j.ajtas.20160501.11

[60] Campbell S Greenwood M Prior S Shearer T Walkem K Young S . Purposive sampling: complex or simple? Research case examples. J Res Nur. (2020) 25:652–661. 10.1177/1744987120927206 PMC7932468 34394687

[61] Hesslinger VM Carbon CC Hecht H. Social Factors in Aesthetics: Social Conformity Pressure and a Sense of Being Watched Affect Aesthetic Judgments. Iperception. (2017) 8:2041669517736322. doi: 10.1177/2041669517736322 29201336 PMC5697602

[62] Parette P Scherer MJ. Assistive Technology Use and Stigma. Education and Training in Developmental Disabilities. (2004) 39:217–226.

[63] Hreha KJ Wong I Molton I Nelson IK Lee D. The Impact of Stroke on Psychological and Physical Function Outcomes in People With Long-Term Physical Disability. Disabil Health J. (2020) 13:100919. doi: 10.1016/j.dhjo.2020.100919 32317243

