# Supplementary material for: Wheelchair as a nexus: understanding stigma in older adults with stroke in China
Source: Front Med (Lausanne). 2026 Jan 13;12:1742686. doi: 10.3389/fmed.2025.1742686 (PMC12834758; doi:10.3389/fmed.2025.1742686)
Supplement: Supplementary file 1 [file Table_1.DOCX]

**Appendix 1: Interview Protocol**

**1.1 Interview Protocol for Older Adults with Stroke**

| **Introduction (5 minutes)** |
| --- |
| Opening remarks/greeting session/ ice-breaking session  (a) Introduction session.  (b) Thank the informant for his/her willingness to share information with the researcher.  (c) Describe the nature of the study and the purpose of the in-depth interviews.  (d) Declare confidentiality and anonymity of the informant. The findings are strictly for academic purposes. Moreover, if required, the use of pseudonyms will be in replacement of names.  (e) Explain that the expected duration of the interview session is 30minutes.  (f) Get the informant's permission to record the interview.  (g) Can you tell me about yourself such as your personal background and professional background? (Name, age, origin) |
| **Research Questions:** What are the probable properties of wheelchairs contributing to the stigmatisation of older adults with stroke? (20 minutes) |
| **RQ1:** What is the stigma perception of older adults with stroke? (10 minutes)  (a) Have you ever had any experiences in your daily life where you felt you were treated differently or received special attention after stroke? How did those experiences make you feel?  (b) Do you ever worry that people may misunderstand your condition after stroke? Has this worry influenced your daily activities or feelings?  (c) In interactions with others after stroke, have you experienced moments where you felt uncomfortable? Could you describe the situation and how you felt about it in detail?  (d) When faced with such situations, how do you typically respond or adjust your emotions and actions?  (e) Since the stroke, have you noticed any changes when participating in social activities or interacting with friends and families? How have these changes affected your life?  **RQ2:** How does the use of a wheelchair influence the stigma perception of older adults with stroke? (10 minutes)  (a) How do you feel about your current wheelchair? Do you find it comfortable and easy to use? Can you share some specific experiences or moments when using it?  (b) Have you noticed any changes in how people interact with you when you are using your wheelchair? Could you describe these situations?  (c) Do you feel that using a wheelchair makes certain social situations easier or harder for you? Could you share some examples of these experiences?  (d) Are there features or aspects of your wheelchair that you think affect how others perceive you or how confident you feel? Can you describe them?  (e) What features of your wheelchair make you feel good about yourself or make interactions with others more comfortable?  (f) If you could improve your wheelchair or design a new one, what features or functions would you add to make it better for your needs? |
| **Conclusion (5 minutes)** |
| (a) Before we conclude, are there any additional questions, thoughts, or experiences you'd like to share that we haven't discussed?  (b) Do you have any feedback about this interview process, or would you like to share any other comments related to our discussion?  (c) With your permission, may l contact you again if we need to clarify any details or if additional questions arise as the study progresses?(d) Would you like a copy of the interview transcript for your review? This allows you to verify the accuracy of the data and ensure your views are correctly represented.  (e)Thank you very much for your valuable time and insights. Once this study is complete, l would be happy to share a summary of the findings with you if you're interested. |

**1.2 Interview Protocol for Stakeholders**

| **Introduction (5 minutes)** |
| --- |
| Opening remarks/greeting session/ ice-breaking session  (a) Introduction session.  (b) Thank the informant for his/her willingness to share information with the researcher.  (c) Describe the nature of the study and the purpose of the in-depth interviews.  (d) Declare confidentiality and anonymity of the informant. The findings are strictly for academic purposes. Moreover, if required, the use of pseudonyms will be in replacement of names.  (e) Explain that the expected duration of the interview session is 30minutes.  (f) Get the informant's permission to record the interview.  (g) Can you tell me about yourself such as your personal background and professional background? (Name, age, origin) |
| **Research Questions:** What are the probable properties of wheelchairs contributing to the stigmatisation of older adults with stroke? (35 minutes) |
| **RQ1:** How is the stigma related to older adults with stroke enacted? (15 minutes)  (a) Do you think people hold negative attitudes or feelings toward older adults with stroke? If so, why do you believe these attitudes exist? Can you provide specific examples or observations?  (b) In your experience, do older adults with stroke exhibit negative feelings toward themselves? If so, could you share examples of how these feelings are reflected in their thoughts, behaviors, or physical responses?  (c)Have you observed older adults with stroke being treated unfairly due to their condition? Could you describe specific instances or situations that highlight this treatment?  **RQ2:** How does wheelchair usage contribute to the stigma perception of older adults with stroke? (20 minutes)  (a)Have you observed that people hold negative attitudes or feelings toward older adults with stroke using wheelchairs? If yes, what do you think causes these attitudes? Can you provide specific examples or situations?  (b) In your experience, what aspects or features of wheelchairs seem to influence discrimination against older adults with stroke? Could you share specific examples or describe these features in detail?  (c) What characteristics or features do you think a wheelchair should have to reduce stigma and enhance the well-being of older adults with stroke? Can you provide examples or describe an ideal wheelchair design? |
| **Conclusion (5 minutes)** |
| 1. Before we conclude, are there any additional questions or insights related to the topics we discussed that you would like to share? 2. Do you have any other comments, feedback, or reflections regarding the interview or the topics we explored today? 3. With your permission, may l contact you again if further clarification or additional insights are needed during the research process? Would you like a copy of the interview transcript? Mention data verification. 4. Would you like a copy of the interview transcript for review? This will allow you to verify the accuracy of the data and provide any corrections or   additional feedback if necessary.   1. Thank you very much for your valuable time and insights. Once this study is complete, l would be happy to share a summary of the findings with you if you're interested. |

**1.3 Interview Protocol for Members of the General Public**

| **Introduction (5 minutes)** |
| --- |
| Opening remarks/greeting session/ ice-breaking session  (a) Introduction session.  (b) Thank the informant for his/her willingness to share information with the researcher.  (c) Describe the nature of the study and the purpose of the in-depth interviews.  (d) Declare confidentiality and anonymity of the informant. The findings are strictly for academic purposes. Moreover, if required, the use of pseudonyms will be in replacement of names.  (e) Explain that the expected duration of the interview session is 30minutes.  (f) Get the informant's permission to record the interview.  (g) Can you tell me about yourself such as your personal background and professional background? (Name, age, origin) |
| **Research Questions:** What are the probable properties of wheelchairs contributing to the stigmatisation of older adults with stroke? (35 minutes) |
| **RQ1:** How is the stigma related to older adults with stroke enacted? (15 minutes)  (a) What are your thoughts or feelings about older adults with stroke? Can you share examples of how people in your community perceive them?  (b) How do you feel about interacting with older adults with stroke? Are there any factors that influence your willingness to engage with them?  (c) In your opinion, how does society view older adults with stroke? Do you think they face any unfair treatment or challenges? Could you provide specific examples?  **RQ2:** How do wheelchairs contribute to the stigma perception of older adults with stroke? (20 minutes)   1. How do you feel when you see older adults with stroke using wheelchairs? What emotions or thoughts come to mind? 2. What are your perceptions of the wheelchairs used by older adults with stroke? Do you think the design or appearance of the wheelchair impacts how others view them? 3. In your opinion, what features or designs of a wheelchair are most helpful for older adults with stroke? Could you provide specific examples? 4. Are there any aspects of wheelchair design that you think might unintentionally contribute to stigma or negative perceptions? Can you provide examples?   (e) Are there specific features or designs of a wheelchair that you think could promote positive perceptions or reduce stigma? Can you share some examples? |
| **Conclusion (5 minutes)** |
| 1. Before we conclude, are there any additional questions or insights related to the topics we discussed that you would like to share? 2. Do you have any other comments, feedback, or reflections regarding the interview or the topics we explored today? 3. With your permission, may l contact you again if further clarification or additional insights are needed during the research process? Would you like a copy of the interview transcript? Mention data verification. 4. Would you like a copy of the interview transcript for review? This will allow you to verify the accuracy of the data and provide any corrections or   additional feedback if necessary.   1. Thank you very much for your valuable time and insights. Once this study is complete, l would be happy to share a summary of the findings with you if you're interested. |
