# Supplementary material for: Wheelchair as a nexus: understanding stigma in older adults with stroke in China
Source: Front Med (Lausanne). 2026 Jan 13;12:1742686. doi: 10.3389/fmed.2025.1742686 (PMC12834758; doi:10.3389/fmed.2025.1742686)
Supplement: Supplementary file 2 [file Table_2.DOCX]

**Appendix 2: Coding tree illustrating theme development**

| **No.** | **Data Source** | **Example of Raw participant quote** | **Initial Codes** | **Sub-themes** | **Final Themes** |
| --- | --- | --- | --- | --- | --- |
| 1 | Members of the general public | “When purchasing a wheelchair from a store, it is typically grouped with medical equipment and not considered a common household item.”(GP4) | Grouped with medical equipment | Symbol of disability | Symbolic Impact on Personal Identity |
| 2 | Members of the general public | “When I see a wheelchair, like something only used in hospitals, I immediately associate the person with illness. It doesn’t feel like an object people would normally use at home or in the community.”(GP6) | Medicalized identity | Symbol of disability | Symbolic Impact on Personal Identity |
| 3 | Members of the general public | “I feel that using a wheelchair amplifies the visible signs of disability and makes their physical limitations more obvious to me.”(GP10) | Visible impairment | Symbol of disability | Symbolic Impact on Personal Identity |
| 4 | Members of the general public/ Older adults with stroke | “Before, I could do many things, but now I cannot do anything except sit in a wheelchair. I would rather stay in a nursing home now; at home, I am stressed.” (SA7) | Burden on family | Symbol of dependence | Symbolic Impact on Personal Identity |
| 5 | Members of the general public | “After the stroke, it seems like many everyday things require help once a wheelchair is involved. Simple activities such as going downstairs or shopping alone appear difficult, and the person seems more reliant on family members.” (GP7) | Dependence in daily life | Symbol of dependence | Symbolic Impact on Personal Identity |
| 6 | Older adults with stroke | “Before my stroke, I was very athletic, and after I retired, I used to ride my bicycle everywhere. Now, I am confined indoors.” (SA6) | Unable to participate in social activities | Symbol of dependence | Symbolic Impact on Personal Identity |
| 7 | Members of the general public | “Even though he has had a stroke, he is still able to move around freely, and he can still go to places where ordinary people go. Well, it is good enough to do things that ordinary people can do without the help of young people.” (GP6) | Regain mobility | Symbol of autonomy | Symbolic Impact on Personal Identity |
| 8 | Members of the general public/ Rehabilitation Therapist | “For him, it is a tool that helps him recover step by step. With the wheelchair, he can practice moving independently and slowly regain confidence in daily activities." (RT3) | Tool for recovery | Symbol of autonomy | Symbolic Impact on Personal Identity |
| 9 | Members of the general public/ Caregiver | “The wheelchair is too big and heavy. When we go outside, it takes up so much space that people immediately notice it, and that makes both of us uncomfortable.”(GP3) | Bulky design | Perceived visual heaviness | Perceived Visual Aesthetics |
| 10 | Members of the general public | “The structural parts of most wheelchairs are made of a bunch of tubular metal pipes spliced together, which makes them complicated and not lightweight.” (GP7) | Visually heavy structure | Perceived visual heaviness | Perceived Visual Aesthetics |
| 11 | Members of the general public/ Nurse | “The manual wheelchairs are primarily grey and black, which makes people feel depressed. ” (GP10) | Grey and black tones | Perceived emotional coldness | Perceived Visual Aesthetics |
| 12 | Members of the general public | “Most wheelchairs look very bulky and heavy. When I see one, I immediately think it must be difficult to push and inconvenient to use, especially in daily environments.” (GP9) | Lack of warmth | Perceived emotional coldness | Perceived Visual Aesthetics |
| 13 | Members of the general public | “The design of the wheelchair gives me a strong medical feeling. It immediately reminds me of illness and disability rather than normal living.” (GP11) | Medical feeling | Perceived emotional coldness | Perceived Visual Aesthetics |
| 14 | Older adults with stroke/ Members of the general public | “When the road surface is uneven, they cannot walk, and they cannot even push. They must rely on us to carry them across, which is too troublesome” (GP4) | Difficult to use outdoors | Environmental adaptability | Adaptability across Daily Living Contexts |
| 15 | Members of the general public/ Rehabilitation Physician | “I often see various manual wheelchairs in stairwells, which occupy much space in our living areas. How can wheelchairs be used both at home and outdoors? That would be more convenient and would not bother other people.” (GP14) | Difficult to use in narrow indoor spaces | Environmental adaptability | Adaptability across Daily Living Contexts |
| 16 | Rehabilitation Physician/ Rehabilitation Therapist | “Many standard wheelchairs have fixed seat widths. For patients with different body sizes or postural changes during rehabilitation, this often leads to discomfort and poor sitting posture.” (PR2) | Seat width cannot be adjusted | Environmental adaptability | Adaptability across Daily Living Contexts |
| 17 | Rehabilitation Physician/ Caregiver | “For many stroke patients, going to the toilet while seated in a wheelchair is difficult. The chair is often too rigid, and transferring in such a small bathroom space becomes stressful, especially when the patient has limited balance.” (PR1) | Difficult for toileting | Functional adaptability | Adaptability across Daily Living Contexts |
| 18 | Nurse/ Caregiver | “Using a wheelchair makes personal care very visible, and many patients feel embarrassed when others are watching.” (RN5) | Privacy issues | Functional adaptability | Adaptability across Daily Living Contexts |
| 19 | Nurse/ Caregiver | “The seat cushion is not comfortable enough for long-term sitting. Some patients complain about pressure and pain after sitting for a while, and we worry about pressure sores if the cushion is not improved.” (CG1) | Seat cushion requires optimization | Functional adaptability | Adaptability across Daily Living Contexts |
| 20 | Members of the general public | “Wheelchairs take up a lot of space at home. In small apartments, it becomes difficult to move around, especially when the wheelchair is parked indoors.” (GP2) | Space-saving | Functional adaptability | Adaptability across Daily Living Contexts |
| 21 | Nurse/ Rehabilitation Therapist | “If the seating posture can be adjusted, patients feel more comfortable and are less likely to complain about pain or stiffness during long periods of sitting.” (RN4) | Adjustable posture | Rehabilitation-stage adaptability | Adaptability across Daily Living Contexts |
| 22 | Rehabilitation Physician/ Rehabilitation Therapist | “The patient needs to maintain the functional position when using the wheelchair, and incorrect postures may lead to external rotation of the legs, which is not conducive to the patient's rehabilitation.” (RT1) | Maintaining functional positioning | Rehabilitation-stage adaptability | Adaptability across Daily Living Contexts |
| 23 | Rehabilitation Physician | “When the wheelchair does not support rehabilitation needs, it actually increases the rehabilitation burden. Patients rely more on staff and family members, and more frustrating for everyone involved.” (PR1) | Rehabilitation burden | Rehabilitation-stage adaptability | Adaptability across Daily Living Contexts |
| 24 | Older adults with stroke/ Members of the general public | “I cannot adjust my wheelchair on my own, and I must ask my caregiver to help me” (SA6) | Restricted independent wheelchair operation | Limited motor control | Autonomy and Control in Wheelchair Use |
| 25 | Rehabilitation Therapist/ Caregiver | “Wheelchair armrests wear out quickly due to frequent use, and replacement parts are hard to find. The armrests also lack adjustability, which is inconvenient.” (CG3) | Handrail design requires optimization | Control-related design limitations | Autonomy and Control in Wheelchair Use |
| 26 | Rehabilitation Physician/ Caregiver | “Older stroke patients often experience leg or foot stiffness, and existing footrests fail to meet their needs. Many seniors resort to removing the footrests.” (CG5) | Pedal design requires optimization | Control-related design limitations | Autonomy and Control in Wheelchair Use |
| 27 | Nurse/ Caregiver | “When the wheelchair is stiff or hard to turn, I have to twist my body awkwardly. After a whole shift, my back feels sore.” (RN4) | Hard to maneuver | Usability for movement | Ease of Operation in Wheelchair Maneuvering |
| 28 | Caregiver | “When I push the wheelchair downhill or need to stop suddenly, the brakes are not easy to control. I have to use a lot of force, and sometimes I worry that I won’t be able to stop it in time, especially when the patient is heavy.” (CG3) | Difficulty in braking | Usability for movement | Ease of Operation in Wheelchair Maneuvering |
| 29 | Rehabilitation Physician / Caregiver | “For many older stroke patients, the footrests do not really meet their needs. Because of limited leg control, they often cannot place their feet securely on the pedals, and during turning, the pedals easily collide with obstacles.” (PR1) | Pedal difficult to use | Ease of movement | Ease of Operation in Wheelchair Maneuvering |
| 30 | Nurse/ Caregiver | “Most of the caregivers in nursing homes were women, and when we encountered heavy older adults, we could not push them at all, and sometimes we could only drag the wheelchair along .” (CG6) | Difficulty in pushing | Ease of movement | Ease of Operation in Wheelchair Maneuvering |
